# GNG2 acts as a tumor suppressor in breast cancer through stimulating MRAS signaling

**DOI:** 10.1038/s41419-022-04690-3

**Published:** 2022-03-23

**Authors:** Anjiang Zhao, Dan Li, Xiongmin Mao, Mengliu Yang, Wuquan Deng, Wenjing Hu, Chen Chen, Gangyi Yang, Ling Li

**Affiliations:** 1 The Key Laboratory of Laboratory Medical Diagnostics in the Ministry of Education and Department of Clinical Biochemistry, College of Laboratory Medicine, Chongqing Medical University, Chongqing, China; 2 Department of Endocrinology, the Second Affiliated Hospital, Chongqing Medical University, Chongqing, China; 3 Department of Endocrinology, Chongqing Emergency Medical Center, Chongqing University Central Hospital, Chongqing, China; 4 Chongqing Prevention and Treatment Hospital for Occupational Diseases, Chongqing, China; 5 Endocrinology, SBMS, Faculty of Medicine, University of Queensland, Brisbane, 4072 Australia

**Keywords:** Breast cancer, Breast cancer

## Abstract

G-protein gamma subunit 2 (GNG2) is involved in several cell signaling pathways, and is essential for cell proliferation and angiogenesis. However, the role of GNG2 in tumorigenesis and development remains unclear. In this study, 1321 differentially expressed genes (DEGs) in breast cancer (BC) tissues were screened using the GEO and TCGA databases. KEGG enrichment analysis showed that most of the enriched genes were part of the PI3K-Akt signaling pathway. We identified GNG2 from the first five DEGs, its expression was markedly reduced in all BC subtype tissues. Cox regression analysis showed that GNG2 was independently associated with overall survival in patients with luminal A and triple-negative breast cancers (TNBC). GNG2 over-expression could significantly block the cell cycle, inhibit proliferation, and promote apoptosis in BC cells in vitro. In animal studies, GNG2 over-expression inhibited the growth of BC cells. Further, we found that GNG2 significantly inhibited the activity of ERK and Akt in an MRAS-dependent manner. Importantly, GNG2 and muscle RAS oncogene homolog (MRAS) were co-localized in the cell membrane, and the fluorescence resonance energy transfer (FRET) experiment revealed that they had direct interaction. In conclusion, the interaction between GNG2 and MRAS likely inhibits Akt and ERK activity, promoting apoptosis and suppressing proliferation in BC cells. Increasing GNG2 expression or disrupting the GNG2–MRAS interaction in vivo could therefore be a potential therapeutic strategy to treat BC.

## Introduction

Breast cancer (BC) is among the most common and deadly types of cancer in women, >1.6 million patients are newly diagnosed with invasive BC in China annually, and BC is associated with considerable physical, emotional, and socioeconomic burdens on the patients and their families, as well as a society [1, 2]. Although breast-conserving surgery or mastectomy followed by adjuvant radiotherapy is currently the standard treatment for breast cancer [3], molecular targeted therapy has attracted considerable attention in this regard. With the development of molecular biology techniques, several molecular targeted drugs have been tested; however, their clinical effects have mostly been unsatisfactory. The identification of reliable tumor markers and novel therapeutic targets to improve the treatment of BC is therefore of great interest.

In recent years, with the advent of the mobile internet and the big data era, the data available on biomolecules has increased considerably. The Cancer Genome Atlas (TCGA) and Gene Expression Omnibus (GEO) have proven to be powerful tools for clinical cancer research and can be used for comprehensive multigenomic analysis. In this study, we identified several differentially expressed genes (DEGs) in BC by using bioinformatics analysis, including Gene Ontology (GO), Kyoto Encyclopedia of Genes and Genomes (KEGG) enrichment analysis, and protein co-expression network analysis. In view of these DEGs, G-protein γ 2 subunit (GNG2), a key molecule, was further screened through expression analysis, literature review, and clinical evaluation.

GNG2 is a subunit of the Gβγ-dimer that forms heterotrimeric G protein with a Gα subunit. Heterotrimeric G protein plays an important role in cell proliferation, differentiation, and angiogenesis, and is a potential molecular target in the treatment of various diseases [4, 5]. As a part of heterotrimeric G protein, GNG2 independently inhibits the proliferation and invasion of human malignant melanoma cells [6, 7]. In addition, big data analysis revealed that GNG2 plays a crucial role in many diseases and biological processes, including IgA nephropathy, Down syndrome, colorectal cancer, and thiopurine metabolism [8–11]. However, these results have not been verified in vitro or in vivo. In this study, we identified, for the first time, GNG2 as a molecular target for the treatment of BC.

## Materials and methods

Detailed Materials and Methods are available in Supplementary Material.

## Results

### Identification of DEGs

**Fig. 1:**
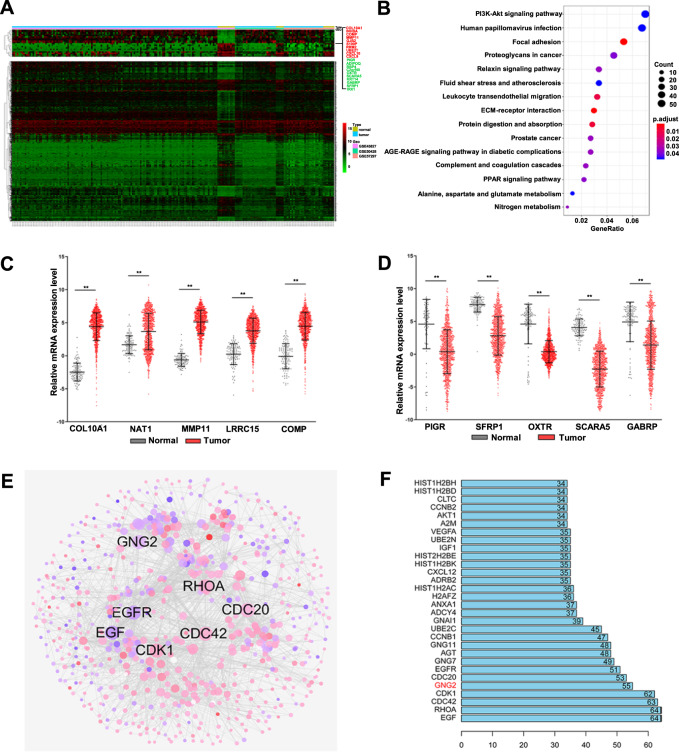
Identification of differential expressed genes (DEGs). **A** Heat maps of the expression levels of DEGs from GEO showed significant differential expression between breast cancer (BC) and normal tissues. **B** KEGG pathway map of the DEGs. **C** The upregulated DEGs obtained from GEO were verified by the TCGA database. **D** The downregulated DEGs obtained from GEO were verified by the TCGA database. **E** Protein-protein interaction (PPI) network constructed with the DEGs. Node size was proportional to its degree. Red nodes represented upregulated genes, and blue nodes represent downregulated genes. **F** The sequence of the top 30 genes with the highest degrees. Error bars indicated SEM. **p < 0.01.

For the conjoint analysis of three expression profile datasets (GSE45827, GSE50428, and GSE57297), we used the R/SVA software package to normalize the batch effect of the conjoint analysis system and remove the interference of batch effect and other unrelated variables in the high-throughput experiment (Fig. S1A and B). By using the aforementioned analysis methods, 1321 DEGs were identified, of which 624 were upregulated and 697 were downregulated. The cluster analysis of DEGs was performed using a heatmap (Fig. 1A). The top 15 upregulated and downregulated genes were shown in Table S4 and S5. To determine the biological features of the DEGs, we performed GO functional enrichment and KEGG pathway analysis. The GO items enriched by DEGs included biological process, cell composition, and molecular function, as shown in Fig. S1C. KEGG analysis showed that most of the DEGs were enriched in the PI3K-Akt signaling pathway (Fig. 1B).

We then used TCGA datasets to verify the results of the GEO analysis. The five upregulated or downregulated DEGs obtained from GEO were consistent with the results obtained from TCGA (Fig. 1C and D), indicating that the results based on the analysis of GEO datasets were reliable.

To further identify key regulatory molecules from DEGs, the correlation between all the DEGs was analyzed using the STRING online database, and an extended co-expression network was constructed using Cytoscape software (Fig. 1E). The network Edges were constructed if two molecules were significantly co-expressed (confidence level > 0.7; *p* < 0.01). In the network, we identified 4,038 pairs of significant co-expression relationships with 836 DEGs.

The degree was used to define the number of interacting proteins around a molecule, the degree of each molecule was then sorted from large to small, and the first 30 molecules with high connectivity degrees were visualized. As shown in Fig. 1F, the first five molecules with the largest degree were EGF (degree 64), RhoA (degree 64), Cdc42 (degree 63), CDK1 (degree 62), and GNG2 (degree 55). These molecules likely play an important role in the development of BC.

### GNG2 expression is downregulated in BC and is related to patient survival rate

**Fig. 2:**
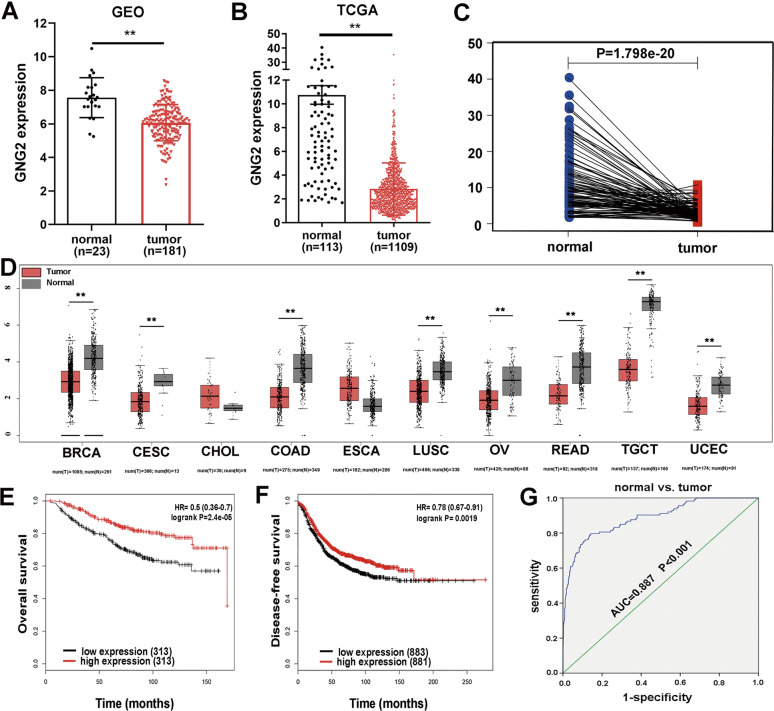
Identification of GNG2 in breast cancer (BC). **A** and **B** GNG2 expression levels were obtained from the GEO database (**A**) and TCGA database (**B**) in normal and BC tissues. **C** The pairing difference analysis of GNG2 expression between tumor and normal tissues. **D** GNG2 expression obtained from GEPIA analysis in multiple cancers including BC, and cervical squamous cell carcinoma (CESC), etc. **E** Kaplan-Meier survival curves for overall survival (OS) according to GNG2 expression levels in BC subjects. **F** Disease-free survival (DFS) curve based on GNG2 expression levels in BC subjects. **G** ROC curve for GNG2 expression in normal and BC tissues. Error bars indicate SEM. **p < 0.01.

We chose GNG2, one of the top five genes with the largest degree, for further study. GEO analysis showed that GNG2 was downregulated in BC (Fig. 2A), which was confirmed in the TCGA database (Fig. 2B). The differential expression of these genes could be directly observed by paired differential analysis (Fig. 2C). In addition, we submitted GNG2 to Gene Expression Profiling Interactive Analysis (GEPIA; http://gepia.cancer-pku.cn/) to detect the difference in gene expression between tumor and normal samples. GNG2 expression was downregulated in multiple cancers, including BC, cervical squamous cell carcinoma (CESC), and colonic adenocarcinoma (COAD; Fig. 2D). Kaplan-Meier survival analysis showed that low GNG2 expression was associated with worse OS and DFS in patients with BC (Fig. 2E and F), while the expression of EGF, RhoA, Cdc42, and CDK1 had no significant effect on OS and DFS (data not shown). Logistic regression analysis revealed that lower GNG2 expression was significantly correlated with higher stage of cancer (OR = 0.616 for stage II vs. I, *p* < 0.01), higher tumor proliferation (OR = 0.584 for > 2 cm vs. ≤ 2 cm; *p* < 0.001), higher tumor status (OR = 0.091; *p* < 0.001), and higher age (OR = 0.681; *p* < 0.001; Table S6). For all functional elements of GNG2, literature-based functional connectivity analysis using the STRING online tool showed that GNG2 also participates in the PI3K-Akt signaling pathway (Fig. S2). Finally, to predict the diagnostic value of GNG2 for BC, we performed the analyses of the ROC curves with TCGA data. As shown in Fig. 2G, the AUC was 0.887 for BC. The cut-off value of GNG2 for predicting BC was 4.527 fragments per kilobase million (FPKM; sensitivity 79.6% and specificity 85.8%). Therefore, GNG2 likely plays an important role in the occurrence and development of BC and could be a novel biomarker for BC.

### GNG2 expression and its relationship with pathological changes in BC subtypes

**Fig. 3:**
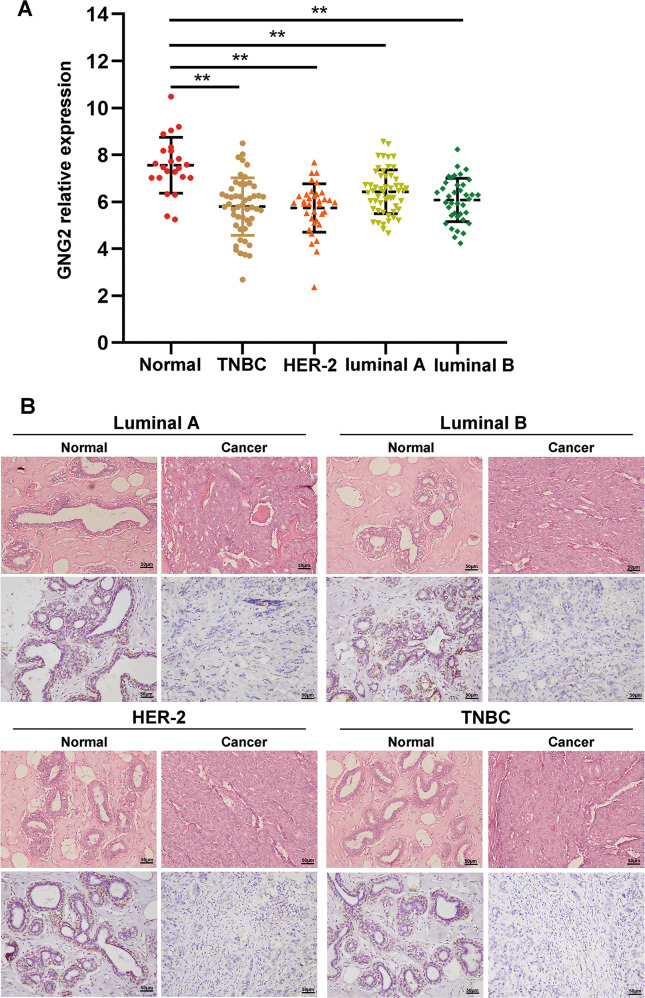
GNG2 expression in BC subtypes. **A** GNG2 mRNA expression in four BC subtypes. **B** Immunohistochemical staining of GNG2 in tumor and non-tumor tissues from patients with luminal A (*n* = 23), luminal B (*n* = 15), HER-2 (*n* = 13), and TNBC (*n* = 12). Data are expressed as the mean ± SD. **p < 0.01.

To identify GNG2 expression in various BC subtypes, we analyzed GEO data and found that GNG2 expression was lower in all four BC subtypes, including the luminal A and B, HER-2, and triple-negative BC (TNBC) than in nontumor tissues, but there was no difference between subtypes of BC (Fig. 3A). In addition, IHC staining also showed that GNG2 expression was significantly lower in all four BC phenotypes than that in nontumor tissues (Fig. 3B).

To determine the impact of GNG2 expression on the prognosis of BC subtypes, the GNG2 mRNA expression was analyzed in clinical samples from the TCGA database. Univariate Cox regression analysis showed that lower GNG2 expression was significantly associated with poorer overall survival in luminal A, HER2, and TNBC subtypes. Meanwhile, multivariate Cox regression analysis found that GNG2 was independently associated with overall survival in patients with luminal A and TNBC (Table S7). These analyses suggest that GNG2 expression may independently predict poor prognosis in patients with the luminal A and TNBC.

### GNG2 inhibits BC cell proliferation in vitro and in vivo

**Fig. 4:**
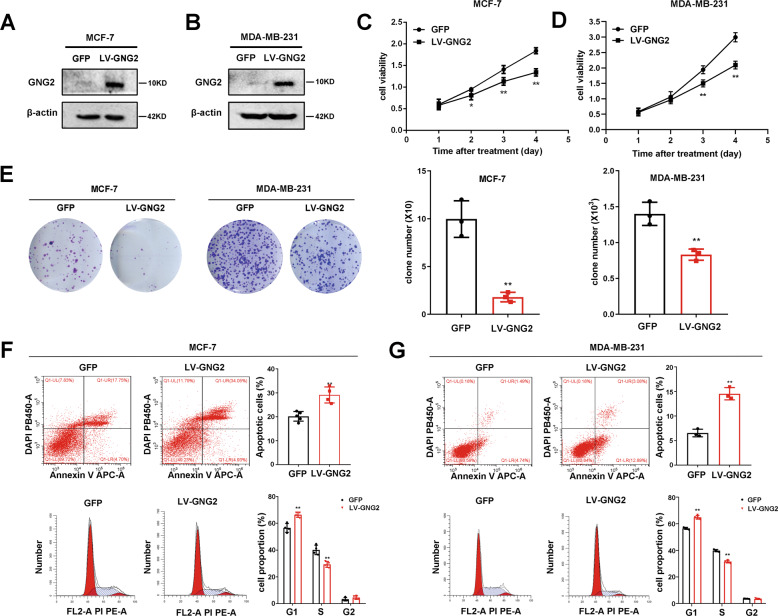
GNG2 inhibits the proliferation of BC cells in vitro. MCF-7 and MDA-MB-231 cells transfected with LV-GFP or LV-GNG2 as described in the Methods. **A** and **B** GNG2 expression at protein levels in MCF-7 (**A**) and MDA-MB-231 (**B**) cells. **C** and **D** Effect of GNG2 overexpression on cell viability in MCF-7 (**C**) and MDA-MB-231 (**D**) cells was detected by CCK-8 assay. **E** Effect of GNG2 overexpression on cell proliferation was determined by colony formation assays (*n* = 3). **F** and **G** Effect of GNG2 overexpression on cell apoptosis and cycle was measured in MCF-7 (**F**) and MDA-MB-231 (**G**) cells by flow cytometry analysis (*n* = 4). Data are expressed as the mean ± SD. * *p* < 0.05, ** *p* < 0.01 vs. GFP.

To elucidate the effect of GNG2 on BC cell proliferation in vitro, MCF-7 and MDA-MB-231 cells were infected with LV-*GNG2*. As expected, GNG2 protein expression markedly increased in these cells (Fig. 4A and B). We used the CCK-8 assay to measure cell viability and found that the upregulation of GNG2 significantly inhibited the proliferation of MCF-7 and MDA-MB-231 cells (Fig. 4C and D). We also evaluated the effect of GNG2 on the proliferation of BC cells by clone formation assay and found that the number of cell clones in the LV-*GNG2*-treated group was significantly less than that in controls (Fig. 4E).

Next, we examined the effect of GNG2 on BC cell apoptosis by using Annexin V-APC-A double staining and flow cytometry analysis. Overexpression of GNG2 in MCF-7 and MDA-MB-231 cells significantly increased the percentage of apoptotic cells. In addition, GNG2 overexpression also affected cell cycle distribution and induced sub-G1 phase arrest (Fig. 4F and G). qRT-PCR and western blot analysis showed that the cell cycle promoter cyclin D1, proliferation marker Ki67, and anti-apoptosis protein Bcl-2 were significantly downregulated in GNG2-overexpressing MCF-7 and MDA-MB-231 cells (Fig. S3A and B).

### GNG2 inhibits BC tumorigenesis in vivo

**Fig. 5:**
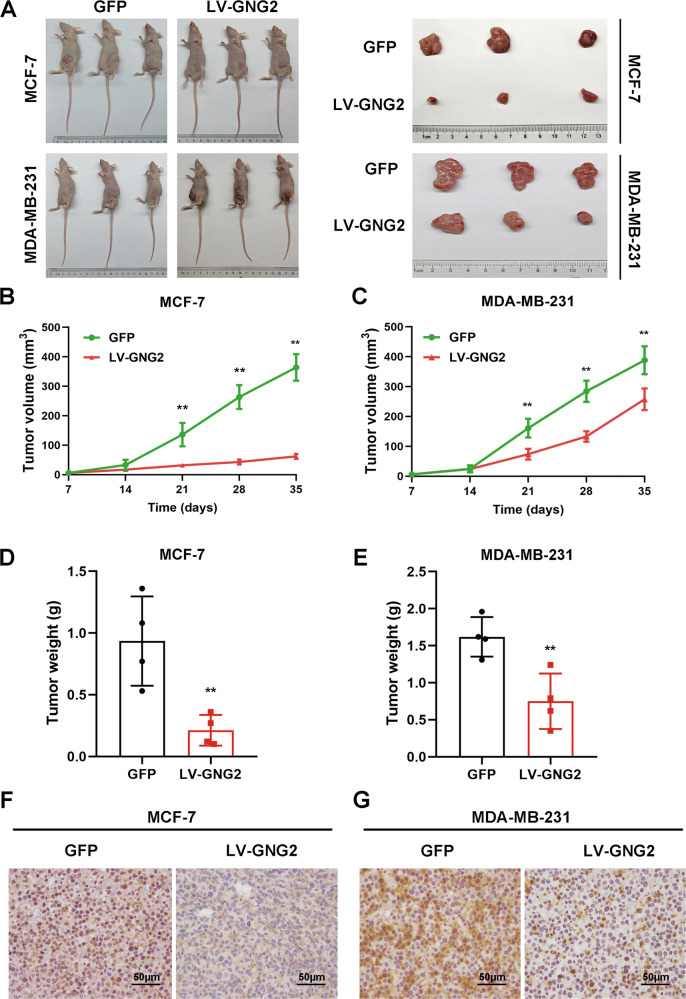
GNG2 inhibits BC tumorigenesis in vivo. Female nude mice were subcutaneously injected with LV-*GNG2*- or LV-*GFP*-infected MCF-7 or MDAMB-231 cells as described in Methods. **A** Photos of the tumor-bearing nude mice and the xenograft tumors dissected from the nude mice. **B** and **C** Tumor growth curves in nude mice injected with MCF-7 (**B**) and MDAMB-231 (**C**) cells. **D** and **E** The weight of xenograft tumors from mice injected with MCF-7 (**D**) and MDA-MB-231 (**E**) cells (*n* = 4). **F** and **G** Immunohistochemical analysis of Ki67 protein in tumor tissues transfected with GNG2-overexpressing MCF-7 (**F**) and MDA-MB-231 (**G**) cells (magnification of 400×), Scale bars, 50 μm. Data are expressed as the mean ± SD. * *p* < 0.05, ** *p* < 0.01 vs. GFP.

We used BC xenograft models to determine the effect of GNG2 on tumorigenesis in vivo. Nude mice were subcutaneously implanted with LV-*GNG2*- or LV-*GFP*- infected MCF-7 or MDA-MB-231 cells. As shown in Fig. 5A, the tumor volume in mice implanted with LV-*GNG2* cells was significantly smaller than that in mice implanted with LV-*GFP* cells 3 weeks after transplantation. In addition, the size and weight of the xenograft tumors from GNG2-overexpressing cells were significantly smaller and lighter than tumors from control cells (Fig. 5B–E). IHC staining also showed that Ki-67 expression was significantly lower in xenograft tumors from GNG2-overexpressing cells than that in the control group (Fig. 5F and G). These data indicated that GNG2 overexpression could inhibit BC growth and proliferation.

### GNG2 overexpression inhibits the activity of ERK and Akt

**Fig. 6:**
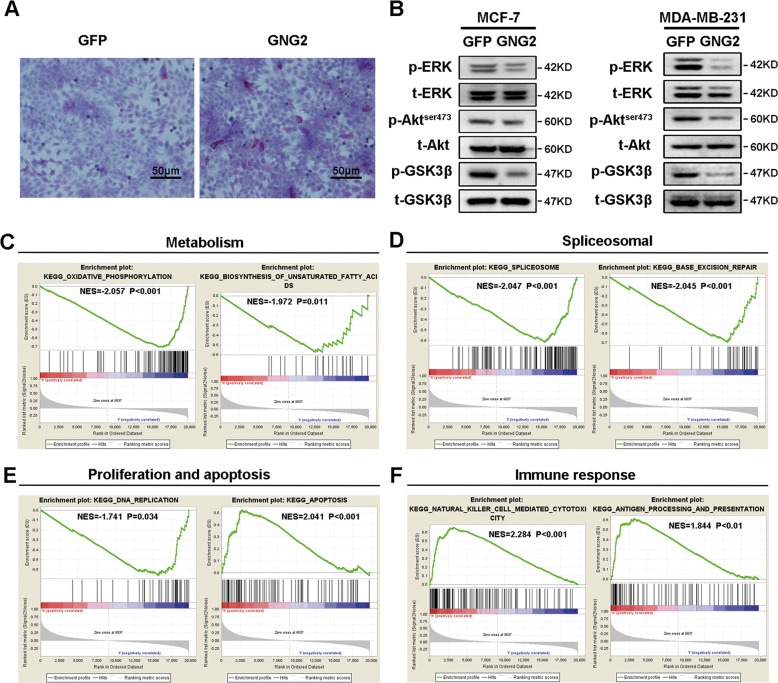
GNG2 is related to multiple signal pathways in breast cancer (BC). MCF-7 and MDA-MB-231 cells were transfected with LV-*GNG2* or LV-*GFP* as described in the Methods. **A** GNG2 overexpression in MCF-7 cells increased glycogen contents, Scale bars, 50 μm. **B** Total and phosphorylated Akt, ERK, and GSK3β in MCF-7 and MDA-MB-231 cells. **C–F** Enrichment plots from gene set enrichment analysis (GSEA). GSEA analyses showing differential enrichment of genes related to oxidative phosphorylation and biosynthesis of unsaturated fatty acids (**C**), spliceosome and base excision repair (**D**), DNA replication and apoptosis (**E**), cytotoxicity mediated by natural killer cell and antigen processing and presentation (**F**) in GNG2-related BC. NES normalized enrichment score.

To elucidate the mechanism by which GNG2 inhibits BC cell proliferation, we studied the effect of GNG2 on energy metabolism and the related signaling pathways. As shown in Fig. 6A, the glycogen content in MCF-7 cells overexpressing GNG2 was significantly higher, than that in cells expressing GFP, suggesting that GNG2 interferes with glycogen mobilization in BC cells. Western blot analysis revealed that the phosphorylation levels of ERK, Akt, and GSK3β in MCF-7 and MDA-MB-231 cells infected with LV-*GNG2* were significantly lower than those in cells infected with LV-*GFP* (Fig. 6B). These data indicated that GNG2 inhibited energy metabolism and the activity of ERK and Akt in BC cells.

### GSEA identifies a GNG2-related signaling pathway

To identify signaling pathways that are activated in BC, a GSEA was performed to compare the low and high GNG2 expression datasets. GSEA revealed that GNG2-related genes were mainly enriched in metabolic, spliceosomal, cell proliferation and apoptosis, and immune response pathways at high or low levels of GNG2 in BC samples (Fig. 6C–F). High GNG2 expression is mainly enriched in apoptosis, natural killer cell-mediated cytotoxicity, antigen processing, and presentation, while low GNG2 expression is mainly involved in DNA replication, oxidative phosphorylation, biosynthesis of unsaturated fatty acid, splicing and spliceosome, and base excision repair. These data indicated that GNG2 could inhibit tumor growth in many ways.

### GNG2 inhibits ERK and Akt activity in an MRAS dependent manner

**Fig. 7:**
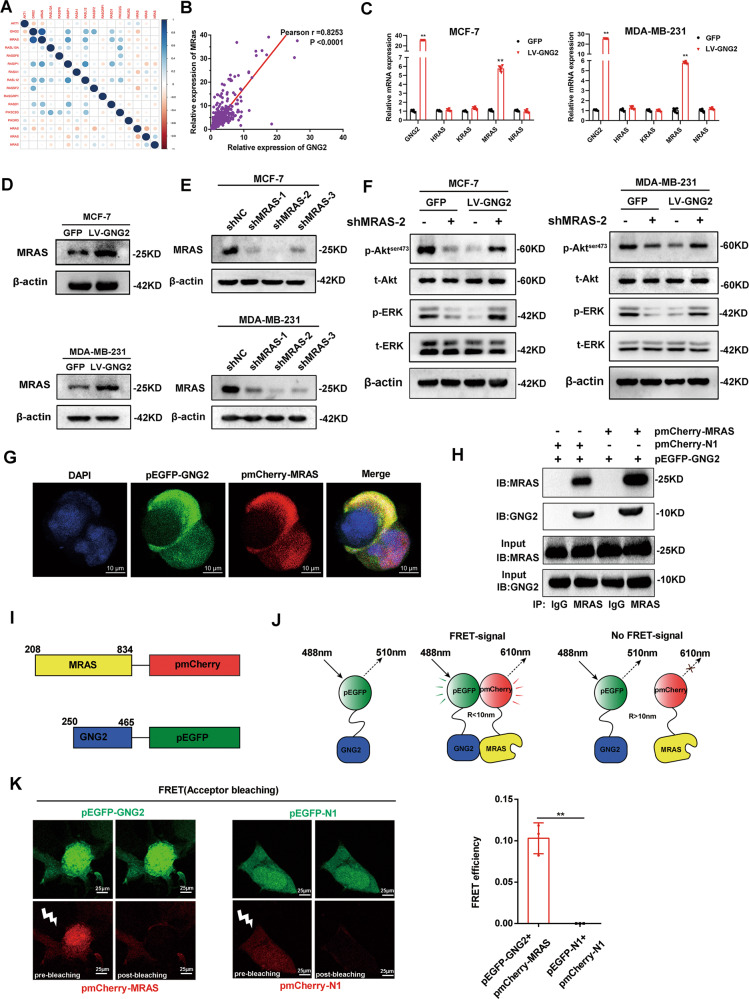
GNG2 inhibits ERK and Akt activity in an MRAS dependent manner. **A** Correlation analysis between GNG2 and RAS family proteins in BC tissues. **B** Correlation between GNG2 and MRAS expression in BC tissues. **C** The mRNA expression of GNG2 and RAS family genes in MCF-7 and MDA-MB-231 cells (*n* = 6). **D** Protein expression of MRAS in GNG2- or GFP-transfected MCF-7 and MDA-MB-231 cells. **E** Transfection efficiency of pGV-shMRAS-1-3 in MCF-7 and MDA-MB-231 cell. **F** Total and phosphorylated Akt and ERK in LV-GNG2 or LV-GFP-transected MCF-7 and MDA-MB-231 cells treated with or without pGV-shMRAS-2. **G** Subcellular localization of GNG2 and MRAS in HEK293T cells was observed by confocal imaging. Scale bars, 10 μm. **H** Co-immunoprecipitation (Co-IP) of GNG2 and MRAS was performed. MCF-7 cells were co-transfected with pEGFP-*GNG2* and pmCherry-*MRAS* or pEGFP-GNG2 and pmCherry-N1 for 48 h. Cell lysates were then collected for immunoprecipitation (IP) with anti-MARS or control IgG antibodies as indicated in the methods. **I** Schematic description of constructs used for FRET assay. The number represents the amino acid residue of the protein. Two fluorescent proteins, GFP and mCherry, were attached to C-terminals of the GNG2 and MRAS proteins, respectively. **J** Schematic representation for the in vitro FRET assay, which measures two-component protein interaction system. GFP-GNG2 was excited by light of 488 nm with a bandwidth of 10 nm (488–10 nm). After excitation, it emited radiation with a longer wavelength of 510–10 nm. When GNG2 interacted with MRAS, the receptor (mCherry) approached GFP closely. FRET signal of 610–10 nm emission was then generated between two fluorescent proteins. R represents the distance between two fluorescent proteins. **K** Fluorescence imaging (left) and efficiency (right) of FRET assay for the interaction between the GNG2 and MRAS in MCF-7 cells. Data are expressed as the mean ± SD. ** *p* < 0.01 vs. GFP.

The Ras protein family plays an important role in the regulation of Akt and ERK activity [12, 13]. We speculated that the members of the Ras family could mediate the effect of GNG2 on the ERK and Akt pathways. Therefore, we utilized the RNA expression data from the TCGA database to analyze the correlation between the differential expression of GNG2 and Ras family proteins in BC. We found a significant positive correlation between GNG2 and MRAS, with a correlation coefficient of 0.82 (Fig. 7A and B). qRT-PCR confirmed that GNG2 could upregulate the mRNA expression of MRAS, but had no significant effect on the expression of NRAS, KRAS, and HRAS in MCF-7 and MDA-MB-231 cells (Fig. 7C). Western blot analysis revealed that MRAS protein expression was significantly upregulated in MCF-7 and MDA-MB-231 cells treated with LV-GNG2 compared to that in cells treated with LV-GFP (Fig. 7D). Subsequently, to determine whether MRAS plays a role in the GNG2-mediated regulation of Akt and ERK activity, we used short hairpin RNAs (shMRAS) to inhibit MRAS expression in vitro. The data presented in Fig. 7E showed that *sh*RNA MRAS fragments markedly reduced the MRAS expression levels, of which *sh*MRAS-2 was the most affected one. As expected, MRAS knockdown almost completely reversed the inhibitory effects of LV-GNG2 on cell viability (Fig. S4A) and proliferation (Fig. S4B). Meanwhile, the effect of GNG2 on the protein expression of proliferation- or apoptosis-related genes was also diminished (Fig. S4C). In addition, as shown in Fig. 7F, MRAS knockdown completely eliminated the inhibitory effects of LV-GNG2 on Akt and ERK phosphorylation in MCF-7 and MDA-MB-231 cells. These results demonstrated that GNG2 inhibited tumor growth and phosphorylation of ERK/Akt through an MRAS-dependent pathway. Furthermore, confocal imaging clearly showed that GNG2 and MRAS were co-localized on the cell membrane (Fig. 7G).

To determine whether GNG2 interacted with MRAS, the Co-IP experiment was performed using pEGFP-*GNG2* and pmCherry-*MRAS* fusion expression plasmid in HEK293T and MCF-7 cells. The Co-IP results confirmed that there was a close interaction between GNG2 and MRAS in these cells (Fig. 7H and Fig. S4D). It was unclear on either direct or indirect interaction by such a close colocalization only. To address this question, a fluorescence resonance energy transfer (FRET) assay was performed between green fluorescent protein (pEGFP-GNG2) and red fluorescent protein (pmCherry-MRAS) (Fig. 7I). A direct interaction between the GNG2 and the MRAS domain brought two fluorescent proteins into a close proximity (approximately 1–10 nm distance) (Fig. 7J). Upon excitation, the FRET efficiency between pEGFP-GNG2 and pmcherry-MRAS was 11%, while no FRET value was detected between pEGFP-N1 and pmCherry-N1 (Fig. 7K). This significant FRET signal between the two fluorescent proteins convincingly demonstrated that there was truly a direct interaction between the GNG2 and MRAS.

## Discussion

BC is the most common cancer in women [14], and the identification of novel specific molecular markers that can be accurately and effectively used in the diagnosis, treatment, or prognosis evaluation of BC is of great scientific and clinical interest. Based on the comprehensive analysis of three expression profiles of GEO datasets, several candidate genes were screened out from a large number of DEGs, and subsequently verified using large sample TCGA datasets, thereby confirming the reliability of the GEO analysis results.

To identify key regulatory genes from a large number of DEGs, we created a network-based inference method, which is an effective strategy for identifying key molecules and has been successfully applied to gene identification [15, 16]. We used a STRING online database to analyze the correlation between DEGs and constructed an extended co-expression network using Cytoscape software. Cluster analysis yielded seven-candidate molecules (degree > 50): EGF, RhoA, CDC42, CDK1, GNG2, CDC20, and EGFR. We used these genes as search sources, focusing on the identification of new key genes. GNG2 was involved in the regulation of most of the upregulated genes and had an independent effect on OS and DFS in patients with BC. We, therefore, identified it as a target gene-related to BC. These results suggest that network-based screening could be of great use in identifying the role of functional genes in tumorigenesis.

The G protein is a heterotrimer composed of α, β, and γ subunits, and is activated by G-protein coupled receptor and separated into Gα and Gβγ dimers. The dissociation of the trimer Gαβγ promotes the deactivation of downstream factors by Gα and Gβγ [17, 18]. GNG2 is a part of the heterotrimeric G-protein gamma subunit and can inhibit the occurrence and development of human malignant melanoma [6, 7]. However, the role of GNG2 in BC, if any has not been reported to date. Because of the heterogeneity of tumorigenesis and development, elucidating the mechanism of action of GNG2 in BC has great clinical significance.

In the current study, based on gene expression profiles and TCGA analysis, we first found that GNG2 expression was significantly downregulated in BC tissue samples. In addition, Kaplan-Meier survival analysis showed that low GNG2 expression was significantly associated with OS and DFS in patients with BC. We, therefore, hypothesized that GNG2 could be a biomarker for the diagnosis, treatment, and prognosis of patients with BC.

Our in vitro studies showed that GNG2 could significantly promote apoptosis and inhibit the proliferation of BC cells. GNG2 also affected the cell cycle distribution and induced G1 phase arrest. In BC cells that overexpressed GNG2, Cyclin D1, Ki67, and Bcl-2 were significantly downregulated. These proteins play important roles in regulating cell proliferation, apoptosis, and cell cycle progression [19–21]. Similarly, in vivo experiments showed that the tumors from MCF-7 and MDA-MB-231 cells overexpressing GNG2 in nude mice were smaller than those in the control group. These results indicate that GNG2 may be an independent indicator for the diagnosis and prognosis of BC.

Tumor cells require a variety of energy sources to support their differentiation and rapid growth [22]. BC cells do not synthesize glycogen; glycogen stored in BC cells is the main energy source for cell proliferation. Therefore, inhibition of glycogen utilization is a key therapeutic strategy for the treatment of BC [23]. Our in vitro study showed that the glycogen content in MCF-7 cells overexpressing GNG2 was significantly higher than that in the control cells. This indicates that GNG2 overexpression may block glycogen utilization and cause energy deficiencies in BC cells, thereby inhibiting their proliferation.

The MAPK and PI3K-Akt signaling pathways play a key role in regulating cell proliferation and differentiation, and their activation promotes the growth of some human tumors [24, 25]. Mammalian MAPK signaling cascades include ERK, c-JNK, and p38. The phosphorylation levels of Akt and ERK increase significantly in many tumors, and Akt and ERK phosphorylation promotes tumorigenesis via a variety of carcinogenic events, including cell proliferation and apoptosis [26]. In this study, we found that GNG2 overexpression significantly inhibited Akt and ERK phosphorylation. This result is similar to that of Yajima et al., who found that GNG2 inhibited Akt activity in human malignant melanoma [6]. We then studied GSK-3β, a downstream molecule of Akt and ERK. GNG2 overexpression caused a decrease in GSK-3β phosphorylation, suggesting that its activity was elevated. It has been reported that GSK-3β activity is reduced in a variety of tumors [27]. Thus, the increase in GSK-3β activity may also have an inhibitory effect on BC. Taken together, these data indicate that GNG2 inhibits Akt and ERK phosphorylation and subsequently activates GSK-3β to inhibit BC cell proliferation and promote apoptosis [28].

In previous studies, overexpression of the Gβγ dimer of G protein promoted cell proliferation and invasion [29]. In this study, overexpression of GNG2, a subunit of the Gβγ dimer, inhibited cell proliferation. This could be because GNG2 is an inhibitory subunit of G protein, similar to Gαi1 or GNG7, as previously reported [30].

MRAS is similar to the classical RAS oncoprotein and has many regulatory effects similar to RAS. It has a unique role in cell differentiation and proliferation and modulates cell polarity regulation [31]. In the current study, GNG2 overexpression inhibited Akt and ERK phosphorylation. and GNG2-mediated regulation of Akt and ERK activity was likely MRAS-dependent. Interestingly, the phosphorylation levels of Akt and ERK were still decreased after MRAS expression was inhibited under normal GNG2 expression. Meanwhile, GNG2 had a positive regulatory effect on MRAS in this experiment. The reason for such “paradoxical” is unknown. Young et al. reported that MRAS acted in combination with SHOC2 to form a binary complex. When MRAS-SHOC2 combined together with PP1 to form a ternary complex, MRAS promoted the activity of ERK and Akt [32, 33]. However, when MRAS-SHOC2 band to SCRIB to form the MARS-SHOC2-SCRIB complex, the activity of ERK and Akt was inhibited, indicating that the effect of MRAS on ERK and Akt was bidirectional [32, 33]. It is speculated that when GNG2 is overexpressed, MRAS expression may increase too, and MARS-GNG2 binary complex formation increases, which subsequently form ternary complexes with SCRIB or PI3K to inhibit the activity of ERK and/or Akt. Such speculation may need further in-depth research to establish.

To further investigate the interaction between GNG2 and MRAS, Co-IP, fluorescence colocalization, and FRET experiments were performed to confirm at different levels that there was truly a direct interaction between GNG2 and MRAS. Therefore, we believe that the GNG2–MRAS interaction is required for GNG2 to regulate Akt and ERK activity and therefore inhibit BC cell proliferation. However, the underlying mechanisms require further study.

Our GSEA analysis showed that GNG2 expression was related to cell metabolism, proliferation, and apoptosis. We confirmed this association in vitro and in vivo. In addition, GNG2 expression was negatively correlated with spliceosome and base excision repair but positively correlated with immune response. Spliceosomes are closely related to the occurrence of human cancers [34, 35]. Depletion of spliceosome components could cause cell cycle defects, thereby inhibiting the growth of cancer cells [36]. We found that GNG2 is related to cytotoxicity, antigen processing, and presentation mediated by natural killer cells, indicating that GNG2 inhibits the proliferation of BC cells by promoting immune response. GNG2 could therefore be an important antitumor gene.

There are some limitations in this study. First, the mechanism by which GNG2 regulates GSK3β activity needs more experimental data. Second, additional data are needed to support that glycogen storage and mitochondrial function can be regulated by GNG2. Therefore, future studies will be needed.

## Conclusion

**Fig. 8:**
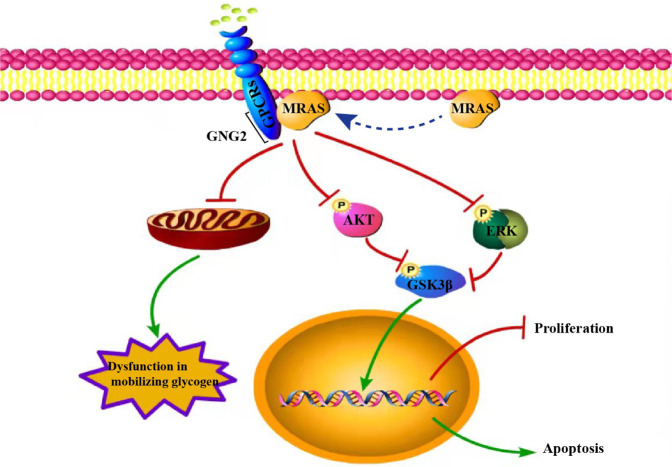
Schematic diagram for the potential mechanisms of GNG2-mediated inhibition of tumorigenesis in BC. Green lines represent promotion, red lines represent inhibition, and dashed lines represent MRAS membrane translocation.

We used a series of bioinformatics analyses and the GEO and TCGA databases to show that GNG2 expression was downregulated in patients with BC. GSEA analysis showed that the expression level of GNG2 was related to cell proliferation and apoptosis. In cellular and animal models, GNG2 overexpression inhibited proliferation and promoted apoptosis of BC cells. The interaction of GNG2 with MRAS inhibits Akt and ERK activity, and thereby promotes BC cell apoptosis and suppresses BC cell proliferation (Fig. 8). Therefore, increasing GNG2 expression or disrupting the GNG2–MRAS interaction in vivo could find application as a novel strategy in the treatment of BC.

### Reporting Summary

Further information on research design is available in the Nature Research Reporting Summary linked to this article.

## Supplementary information

SUPPLEMENTAL MATERIAL

Original Western Blots

Checklist-Reporting Summary

## Data Availability

The datasets used and/or analyzed during the current study are available from the corresponding author on reasonable request.
