# Supplementary material for: GNG2 acts as a tumor suppressor in breast cancer through stimulating MRAS signaling
Source: Cell Death Dis. 2022 Mar 23;13(3):260. doi: 10.1038/s41419-022-04690-3 (PMC8943035; doi:10.1038/s41419-022-04690-3)
Supplement: Supplementary file 1 — SUPPLEMENTAL MATERIAL [file 41419_2022_4690_MOESM1_ESM.pdf]

## Materials and methods

### Data acquisition

GSE45827, GSE50428, and GSE57297 expression profiles were downloaded from the GEO database (https://www.ncbi.nlm.nih.gov/geo/), a publicly available database for high-throughput gene expression analysis. The raw data of gene expression in BC and corresponding clinical information were downloaded from the TCGA official website (https://portal.gdc.cancer.gov/). A total of 1109 breast cancer (BC) specimens and 121 normal breast specimens were included. The clinical characteristics of these samples were shown in Table S1.

### Data processing

Raw data downloaded from GEO were merged using R software. There were 181 BC samples and 23 normal controls after samples with incomplete information were excluded. Samples sequenced from different platforms were normalized using R/SVA packages [1]. The DEGs of patients with BC were extracted using empirical Bayes *t*-tests provided by the R/Limma package. DEGs with [log Fold Change] ≥ 1.5 (adjust $p < 0.01$) were selected for analysis. Heat map of DEGs was drawn using the R/Pheatmap package. Gene expression and clinical raw data downloaded from TCGA were decompressed and merged into a matrix file by Perl software.

### Functional and pathway enrichment analysis

GO and KEGG pathway enrichment analysis of DEGs was performed using the ClusterProfiler R package. GO and KEGG pathway terms were identified with a strict cutoff of $P < 0.05$ and a false discovery rate (FDR) of less than 0.05. After extracting the expression of the GNG2 gene from DEGs by R software, the samples were divided into low and high expression groups divided by the median value of GNG2 expression. A Gene Set Enrichment Analysis (GSEA) software was used for gene set enrichment analysis. All results were visualized by R software.

**Protein-protein interaction (PPI) network analysis**

The PPI network based on all DEGs was constructed using the STRING online database (interaction score > 0.7) and visualized by Cytoscape software. Cytoscape software was also used to make the statistical analysis of nodes, edges, and degrees, and R software was used to visualize the statistical results.

**Cell culture and treatment**

MCF-7 and MDA-MB-231 human breast carcinoma cell lines (kindly provided by Dr. Tingmei Chen, Chongqing Medical University, China) were cultured in DMEM supplemented with 10% fetal bovine serum (Gibco-Invitrogen, Grand Island, NY) and 1% penicillin-streptomycin at 37 ℃ in a humidified atmosphere containing 5% $CO_2$. When cells reached 20 - 30% confluence, they were transfected with lentivirus expressing GNG2 (LV-*GNG2*), or green fluorescence protein (LV-*GFP*) for 72 h. For inducible knockdowns of MRAS, plasmids expressing *sh*RNA against MRAS (pGV-*shMRAS-1-3*) were constructed. Cells were transfected with pGV-*shMRAS-1, 2, 3,* or with the negative controls (pGV248) for 48 h. The sequences were as follows: 5'-CCACCTCTCAATGTCGACAAA-3' for pGV-*shMRAS-1*, 5'-GCGTCAAAGACAGGGAGTCAT-3' for pGV-*shMRAS-2,* and 5'-CAA ACTGCAATGTGTGATCTT-3' for pGV-*shMRAS-3.*

**Cell proliferation and colony formation assay**

For cell proliferation, cells were seeded into 96-well plates at a density of 2000 cells per well. At 0, 24, 48, 72, and 96 h after seeding, cell viability was determined by the cell counting kit-8 (CCK-8) Detection kit (Bimake, Inc. USA) according to the manufacturer's instructions. For colony formation assay, transfected cells were seeded in 6-well plates with 400 cells per well, and cultured for 10 to 14 days. Cultured cells were fixed with methanol for 6 min and stained with crystal violet (Sigma-Aldrich, Inc. USA) for 5-7 min. Colony numbers were determined using Image J.

**Flow cytometry analysis**

MCF-7 and MDA-MB-231 cells were seeded and cultured in 6-well plates for 48 h at 37 ℃,

and then were transfected with LV-*GNG2* or LV-*GFP* for 72 h. Cells were collected and washed in ice-cold PBS. Subsequently, cells were stained with Annexin V APC-A and DAPIPB450-A in Apoptosis Detection kit (BD Biosciences, In. USA) according to the manufacturer's instructions. For determining the cell cycle phase, the cells were fixed in 75% ice-cold ethanol overnight and then treated with 1 mg/ml RNase (Sigma-Aldrich, Inc. USA) for 10 min at 37℃. DNA was stained with propidium iodide (15 mg/ml) for 20 min at 4 ℃ in the dark. Cell cycle profiles and cell apoptosis were analyzed by flow cytometry (FACS Vantage SE, BD, NJ, USA). All experiments were repeated three times.

**Immunohistochemistry (IHC), glycogen staining, and Hematoxylin-eosin (**H&E**) staining**

Paraffin-embedded tissue sections were stained with H&E as previously described [2]. MCF-7 cells were stained with Periodic Acid-Schiff (PAS) using a glycogen staining kit (Solarbio, Beijing, China), according to the manufacturer's instructions. IHC was performed in paraffin-embedded tissue sections following the standard protocol described previously [2]. The slides were blocked with 3% bovine serum albumin (BSA) and incubated with primary antibody (anti-Ki67 or anti-GNG2) at 4 ℃ overnight. Then, biotinylated goat anti-rabbit immunoglobulin was used on the section and incubated for 30 min at 37 ℃. Finally, sections were developed with diaminobenzidine for observation.

**Tumor formation assay *in vivo***

LV-*GNG2*- or LV-*GFP*-infected MCF-7 and MDA-MB-231 cells were injected subcutaneously into the groin of 5-weeks-old female BALB/c-nu athymic nude mice ($1\times10^5$ cells per mouse). The mice were randomly divided into four groups with 4 mice in each group. The longest diameter and widest vertical width of the tumor were examined every 2 days with a dial-caliper. Tumor volume was calculated by the following formula: $L\times W^2\times1/2$. In the 5$^{th}$ week, the mice were killed and the tumor tissues were dissected, photographed, weighed, isolated, sectioned, and stained. Animal experiments were approved by the Animal Ethics Committee of the Chongqing Medical University.

**Survival analysis**

Kaplan–Meier plotter database (http://kmplot.com/analysis/) was used to analyze the relationship between gene expression (GNG2, EGF, RhoA, Cdc42, and CDK1) and survival rate in patients with BC including overall survival (OS) and disease-free survival (DFS) [3].

**Real-time quantitative PCR (RT-qPCR) and Western blots**

qRT-PCR measurements were performed as previously described [2]. The primer pairs were listed in Table S2. β-actin was used as a control gene. Protein analysis was performed with western blots as described previously [2]. The primary antibodies included anti-GNG2 (Abcam, UK, ab198225), anti- muscle RAS oncogene homolog (MRAS) (Abcam, UK, ab176570), anti-marker of proliferation Ki-67 (KI-67) (Abcam, UK, ab16667), anti-B-cell lymphoma-2 (BCL-2) (Cell Signaling Technology, USA, #3498), anti-CyclinD1 (a cell cycle promoter) (Cell Signaling Technology, USA, #2922), anti-Akt/phospho-Akt (Cell Signaling Technology, USA, #9272/#9271), anti-extracellular signal-regulated kinase (ERK)/phospho-ERK (Cell Signaling Technology, USA, #4695/#4376), anti-glycogen synthase kinase 3β (GSK3β)/phospho-GSK3β (Cell Signaling Technology, MA, USA) (Cell Signaling Technology, USA, #5676/#9322), and β-actin (ZSGB-BIO, China, TA-09).

**Co-localization assay**

HEK293T cells were co-transfected with pEGFP-*GNG2* and pmCherry-*MRAS* for 48 h. The sequence of plasmid was shown in Table S3. Cells were seeded and cultured in 12-well plates and fixed with 4% paraformaldehyde for 30 min, followed by three washes of PBS for 5 min each. The cells were counterstained with DAPI for 10 min at room temperature (RT) and then photographed using a confocal microscope (Leica TCS SP8, Germany).

**Co-immunoprecipitation *(Co-IP)***

Co-IP experiment was performed as previously described [4]. In brief, HEK293T and MCF-7 cells were transfected with pEGFP-*GNG2* or pmCherry-*MRAS* alone or together for 48 h. Cells were harvested and lysed in IP buffer with protease inhibitors on ice for 20 min. The cell suspension was centrifuged, and the supernatant was collected, followed by incubation

with re-suspended protein G/A-magnetic beads and anti-MARS or IgG antibody for 60 min at RT with gentle rocking. The coated magnetic beads were incubated with cell lysates overnight at 4 ℃. The magnetized beads were washed, and proteins were immunoblotted using an anti-GNG2-antibody (Novus, USA, H00054331-M03).

**Fluorescence resonance energy transfer (FRET) assay**

MCF-7 cells were co-transfected with the pEGFP-GNG2 and pmCherry-MRAS for 48 h. Cells were then seeded and cultured in a 35 mm confocal microscopy dish and fixed with 4% paraformaldehyde for 30 min. The acceptor bleaching method was used to detect FRET efficiency [5]. Briefly, view field was zooming into cells of interest, highlighting an ROI (region of interest) in which the photo-destruction of the acceptor (mCherry) occurred and then the control recording program began. For photo-destruction of the acceptor, cells were photo-bleached with a 552 nm laser line (set at 10% intensity, 30 times). The images were captured in both channels before and after the photo was bleached. In all experiments, approximately 6-10 cells were measured, and FRET efficiency was calculated as E = (1-pre/post) × 100%, where Pre and Post represented the fluorescence intensity of the donor (GFP) before and after photo-bleaching. Images were observed by a confocal laser microscope (Leica TCS SP8, Germany) with LAS ×FRET AB module.

**Human samples**

This study was performed with approval from the Ethics Committee at Chongqing Medical University. No consent from the patients was needed, and data were analyzed anonymously. A total of 63 pairs of Paraffin-embedded BC samples, including 23 luminal A, 15 luminal B, 13 HER-2, and 12 triple-negative breast cancers (TNBC), were collected at the Chongqing University Cancer Hospital. The GNG2 expression level was examined by IHC.

**Statistical analysis**

Data were expressed as the mean ± SD or SE. GraphPad Prim software was used to drawbar charts and line charts. Statistical analysis and receiver operating characteristic (ROC) curve were performed using SPSS standard version (version 21.0). The colony counts were

performed using the Image J software. Significant differences among multiple groups were assessed by a two-way ANOVA with a post hoc test. A two-tailed unpaired Student's *t*-test was used for comparison between two groups. Differences were considered to be statistically significant at $p < 0.05$.

## References


1. Lindahl GE, Stock CJ, Shi-Wen X, Leoni P, Sestini P, Howat SL, et al. Microarray profiling reveals suppressed interferon stimulated gene program in fibroblasts from scleroderma-associated interstitial lung disease. Respir Res. 2013;14:80.
2. Li X, Yang M, Wang H, Jia Y, Yan P, Boden G, et al. Overexpression of JAZF1 protected ApoE-deficient mice from atherosclerosis by inhibiting hepatic cholesterol synthesis via CREB-dependent mechanisms. Int J Cardiol. 2014;177:100-10.
3. Wang S, Huang G, Hu Q, Zou Q. A network-based method for the identification of putative genes related to infertility. Biochim Biophys Acta. 2016;1860:2716-24.
4. Lai Y, Zhao A, Tan M, Yang M, Lin Y, Li S, et al. DOCK5 regulates energy balance and hepatic insulin sensitivity by targeting mTORC1 signaling. EMBO Rep. 2020;21:e49473.
5. Pfab A, Bruckmann A, Nazet J, Merkl R, Grasser KD. The Adaptor Protein ENY2 Is a Component of the Deubiquitination Module of the Arabidopsis SAGA Transcriptional Co-activator Complex but not of the TREX-2 Complex. J Mol Biol. 2018;430:1479-94.

**Table S1** Clinic characteristics of patients with BC in TCGA

| Clinical characteristics | | Total | % |
|---|---|---|---|
| Age at diagnosis (yr) | | 1097 (26-90) | |
| Stage | I | 183 | 18.0 % |
| | II | 621 | 61.3 % |
| | III | 189 | 18.7 % |
| | IV | 20 | 20.0 % |
| Primary tumor | ≤ 2cm | 916 | 83.7 % |
| | > 2cm | 178 | 16.3 % |
| Lymph nodes | Negative | 515 | 47.9 % |
| | Positive | 561 | 52.1 % |
| Distant metastasis | Negative | 896 | 97.9 % |
| | Positive | 19 | 2.1 % |
| Estrogen receptor | Negative | 238 | 22.8 % |
| | Positive | 808 | 77.2 % |
| Progesterone receptor | Negative | 344 | 33.0 % |
| | Positive | 699 | 67.0 % |
| HER-2 | Negative | 532 | 85.5 % |
| | Positive | 90 | 14.5 % |
| Menopause | Yes | 705 | 75.5 % |
| | No | 229 | 24.5 % |
| Status | With tumor | 1109 | 90.7 % |
| | Tumor free | 113 | 9.3 % |

**Table S2** The primer sequence used for qRT-PCR analysis

| Species | Primer | Sequence (5′-3′) |
|---|---|---|
| Homo sapiens | CyclinD1-F | ACGCTACTATAAAGAGAAGACGAA |
| | CyclinD1-R | AACCAGCATCTCATAAACAGG |
| Homo sapiens | BCL2-F | CTCGTCCAAGAATGCAAAGCAC |
| | BCL2-R | TCTCCCGGTTATCGTACCCTG |
| Homo sapiens | KI67-F | AGAAGGCAACACTACTACAACA |
| | KI67-R | GTTCTGTATCAGGCAAGCTCT |
| Homo sapiens | HRAS-F | ATGACGGAATATAAGCTGGTGGT |
| | HRAS-R | GGCACGTCTCCCCATCAATG |
| Homo sapiens | KRAS-F | ACAGAGAGTGGAGGATGCTTT |
| | KRAS-R | TTTCACACAGCCAGGAGTCTT |
| Homo sapiens | MRAS-F | TTCCTCATCGTCTACTCCGTC |
| | MRAS-R | AGGATCATCGGGAATGACTCC |
| Homo sapiens | NRAS-F | TGAGAGACCAATACATGAGGACA |
| | NRAS-R | CCCTGTAGAGGTTAATATCCGCA |
| Homo sapiens | GNG2-F | CAGCATAGCACAAGCCAGGAA |
| | GNG2-R | GGAACAGGGGTCAGGAGGG |

**Table S3** The sequence of plasmid expressing GNG2 and MRAS

| Gene | mRNA | Plasmid | Sequence (CDS) |
|---|---|---|---|
| GNG2 | NM_053064.5 | pEGFP-GNG2 | 241 agcactccg**a tggccagcaa caacaccgcc agcatagcac aagccaggaa gctggtagag**<br>**301 cagcttaaga tggaagccaa tatcgacagg ataaaggtgt ccaaggcagc tgcagatttg**<br>**361 atggcctact gtgaagcaca tgccaaggaa gaccccctcc tgacccctgt tccggcttca**<br>**421 gaaaacccgt ttagggagaa gaagtttttc tgtgccatcc tttaa**gtctt tgagaggggc |
| MRAS | NM_001085049.3 | pmCherry-MRAS | 181 gagcgcgcgg tctgacctac gagaaac**atg gcaaccagcg ccgtccccag tgacaacctc**<br>**241 cccacataca agctggtggt ggtgggggat gggggtgtgg gcaaaagtgc cctcaccatc**<br>**301 cagtttttcc agaagatctt tgtgcctgac tatgacccca ccattgaaga ctcctacctg**<br>**361 aaacatacgg agattgacaa tcaatgggcc atcttggacg ttctggacac agctgggcag**<br>**421 gaggaattca gcgccatgcg ggagcaatac atgcgcacgg gggatggctt cctcatcgtc**<br>**481 tactccgtca ctgacaaggc cagctttgag cacgtggacc gcttccacca gcttatcctg**<br>**541 cgcgtcaaag acagggagtc attcccgatg atcctcgtgg ccaacaaggt cgatttgatg**<br>**601 cacttgagga agatcaccag ggagcaagga aaagaaatgg cgaccaaaca caatattccg**<br>**661 tacatagaaa ccagtgccaa ggacccacct ctcaatgtcg acaaagcctt ccatgacctc**<br>**721 gttagagtaa ttaggcaaca gattccggaa aaaagccaga agaagaagaa gaaaaccaaa**<br>**781 tggcggggag accgggccac aggcacccac aaactgcaat gtgtgatctt gtga**caggcc |

**Table S4** Major up-regulated genes

| Gene | logFC | *p*-value | Adjusted *p*-value |
|---|---|---|---|
| COL10A1 | 5.3979921 | 1.72E-26 | 3.12E-24 |
| S100P | 4.5675393 | 7.54E-12 | 1.08E-10 |
| MMP11 | 4.3239767 | 1.39E-22 | 1.44E-20 |
| CXCL10 | 4.2492650 | 1.28E-14 | 3.10E-13 |
| RRM2 | 3.8121373 | 2.48E-15 | 1.65E-13 |
| CXCL9 | 3.7138976 | 3.06E-16 | 2.78E-14 |
| INHBA | 3.6970626 | 2.33E-16 | 2.24E-14 |
| GJB2 | 3.6943391 | 8.57E-10 | 1.12E-08 |
| COMP | 3.6198141 | 2.20E-21 | 1.37E-18 |
| UBE2T | 3.5041486 | 6.97E-13 | 2.29E-11 |
| LRRC15 | 3.4906308 | 2.01E-18 | 4.11E-16 |
| GPRC5A | 3.4670335 | 8.14E-20 | 2.64E-17 |
| TPX2 | 3.4579777 | 2.10E-17 | 2.96E-15 |
| PRC1 | 3.4540162 | 2.37E-22 | 3.53E-19 |
| CTHRC1 | 3.4276536 | 2.36E-08 | 2.03E-07 |

T**able S5** Major down-regulated genes

| Gene | logFC | *p*-value | Adjusted *p*-value |
|---|---|---|---|
| PIGR | -7.198453 | 6.50E-30 | 2.06E-27 |
| ADIPOQ | -5.345462 | 2.91E-20 | 2.04E-18 |
| SFRP1 | -5.160778 | 9.64E-16 | 2.92E-14 |
| OXTR | -5.145463 | 1.37E-36 | 1.20E-33 |
| C2orf40 | -4.941419 | 1.51E-23 | 1.92E-21 |
| SCARA5 | -4.895690 | 2.27E-49 | 1.69E-45 |
| IRX1 | -4.839582 | 2.26E-22 | 2.68E-20 |
| GABRP | -4.832302 | 5.08E-10 | 5.09E-09 |
| RBP4 | -4.778688 | 4.25E-29 | 1.20E-26 |
| KRT14 | -4.572475 | 2.11E-11 | 2.78E-10 |
| PLIN1 | -4.519448 | 3.54E-22 | 3.52E-20 |
| LEP | -4.470244 | 4.60E-29 | 1.27E-26 |
| PLIN4 | -4.379155 | 3.45E-27 | 6.77E-25 |
| KRT15 | -4.332337 | 6.86E-17 | 2.49E-15 |
| TGFBR3 | -4.211514 | 3.15E-20 | 2.18E-18 |

**Table S6** GNG2 expression associated with clinical-pathological characteristics (logistic regression).

| Clinical characteristics | Total (N) | Odds ratio in GNG2 expression | *p*-value |
|---|---|---|---|
| Stage (II *vs.* I) | 800 | 0.616 (0.439-0.859) | **0.004** |
| Stage (III *vs.* I) | 428 | 0.799 (0.542-1.176) | 0.256 |
| Stage (IV *vs.* I) | 201 | 0.472 (0.177-1.196) | 0.118 |
| Primary tumor (>2cm *vs.* ≤2cm) | 1094 | 0.584 (0.442-0.769) | **<0.001** |
| Lymph nodes (positive *vs.* negative) | 1076 | 0.900 (0.682-1.187) | 0.457 |
| Distant metastasis (positive *vs.* negative) | 915 | 0.565 (0.224-1.333) | 0.203 |
| Status (with tumor *vs.* tumor free) | 1222 | 0.091 (0.046-0.165) | **<0.001** |
| Age (continue) | 1097 | 0.681 (0.670-0.691) | **<0.001** |

*p* value was less than 0.05, with statistical significance.

**Table S7** Cox regression analysis of characteristics associated with survival of BC subtypes in TCGA.

| Clinicopathologic variable | Univariate Cox regression analysis | | Multivariate Cox regression analysis | |
|---|---|---|---|---|
| | HR (95%CI) | *p*-Value | HR (95%CI) | *p*-Value |
| Luminal A | | | | |
| Primary tumor | 1.147 (0.741-1.776) | 0.538 | 0.803 (0.461-1.396) | 0.437 |
| Lymph nodes (positive *vs.* negative) | 1.458 (0.769-2.763) | 0.247 | 1.588 (0.664-3.795) | 0.298 |
| Distant metastasis (positive *vs.* negative) | 8.563 (2.584-28.37) | < 0.001 | 4.509 (0.821-24.752) | 0.008 |
| Stage (IV *vs.* III *vs.* II *vs.* I) | 1.423 (0.924-2.191) | 0.109 | 1.298 (0.581-2.901) | 0.525 |
| Age (continue) | 1.051 (1.023-1.079) | < 0.001 | 1.052 (1.014-1.091) | 0.005 |
| Menopause (no *vs.* yes) | 3.042 (1.186-7.804) | 0.021 | 1.205 (0.361-4.021) | 0.762 |
| GNG2 (high *vs.* low) | 0.536 (0.339-0.874) | 0.011 | 0.761 (0.525-0.817) | 0.039 |
| Luminal B | | | | |
| Primary tumor | 3.803 (1.937-7.465) | < 0.001 | 2.362 (1.019-5.477) | 0.045 |
| Lymph nodes (positive *vs.* negative) | 11.343 (1.463-87.96) | 0.020 | 2.422 (0.216-27.1) | 0.473 |
| Distant metastasis (positive *vs.* negative) | 5.469 (1.669-17.921) | 0.005 | 0.839 (0.149-4.712) | 0.842 |
| Stage (IV *vs.* III *vs.* II *vs.* I) | 4.613 (2.337-9.104) | < 0.001 | 3.786 (1.128-12.71) | 0.031 |
| Age (continue) | 1.021 (0.977-1.068) | 0.348 | 1.046 (0.991-1.105) | 0.104 |

**Table S7** Continued

| Clinicopathologic variable | Univariate Cox regression analysis | | Multivariate Cox regression analysis | |
|---|---|---|---|---|
| | HR (95%CI) | *p*-Value | HR (95%CI) | *p*-Value |
| Menopause (no *vs.* yes) | 1.059 (0.339-3.309) | 0.921 | 1.281 (0.217-7.546) | 0.783 |
| GNG2 (high *vs.* low) | 0.753 (0.605-1.099) | 0.063 | 0.774 (0.375-0.915) | 0.186 |
| HER-2 | | | | |
| Primary tumor | 2.283 (1.298-4.105) | 0.004 | 1.721 (0.825-3.592) | 0.147 |
| Lymph nodes (positive *vs.* negative) | 1.752 (1.091-2.813) | 0.020 | 1.306 (0.642-2.657) | 0.461 |
| Distant metastasis (positive *vs.* negative) | 0.838 (0.183-3.843) | 0.821 | 0.609 (0.085-4.332) | 0.621 |
| Stage (IV *vs.* III *vs.* II *vs.* I) | 2.543 (1.354-4.775) | 0.003 | 1.357 (0.376-4.897) | 0.641 |
| Age (continue) | 1.074 (1.032-1.117) | < 0.001 | 1.111 (1.044-1.183) | < 0.001 |
| Menopause (no *vs.* yes) | 1.697 (0.604-4.771) | 0.316 | 0.192 (0.032-1.152) | 0.071 |
| GNG2 (high *vs.* low) | 0.372 (0.195-0.769) | 0.006 | 0.695 (0.576-0.864) | 0.098 |
| TNBC | | | | |
| Primary tumor | 3.425 (2.001-5.862) | < 0.001 | 1.893 (0.920-3.893) | 0.083 |
| Lymph nodes (positive *vs.* negative) | 4.297 (2.708-6.818) | < 0.001 | 2.337 (1.247-4.379) | 0.008 |
| Distant metastasis (positive *vs.* negative) | 9.303 (2.117-40.891) | 0.003 | 0.911 (0.075-10.929) | 0.941 |

**Table S7** Continued

| Clinicopathologic variable | Univariate Cox regression analysis | | Multivariate Cox regression analysis | |
|---|---|---|---|---|
| | HR (95%CI) | *p*-Value | HR (95%CI) | *p*-Value |
| Stage (IV *vs.* III *vs.* II *vs.* I) | 5.485 (3.247-9.266) | < 0.001 | 3.784 (0.905-15.825) | 0.068 |
| Age (continue) | 0.998 (0.964-1.034) | 0.936 | 0.974 (0.922-1.029) | 0.359 |
| Menopause (no *vs.* yes) | 1.193 (0.504-2.824) | 0.687 | 2.578 (0.612-10.857) | 0.197 |
| GNG2 (high *vs.* low) | 0.447 (0.285-0.719) | <0.001 | 0.682 (0.484-0.838) | 0.034 |

IHC surrogate was used for molecular subtyping, defined as luminal A (ER and PR+, HER2-), luminal B (ER and PR+, HER2+), HER-2 (ER+, PR+, HER2+), Triple-negative breast cancer (TNBC) (ER-, PR-, HER2-). *p*-value was less than 0.05, with statistical significance.

## Figure S Legends

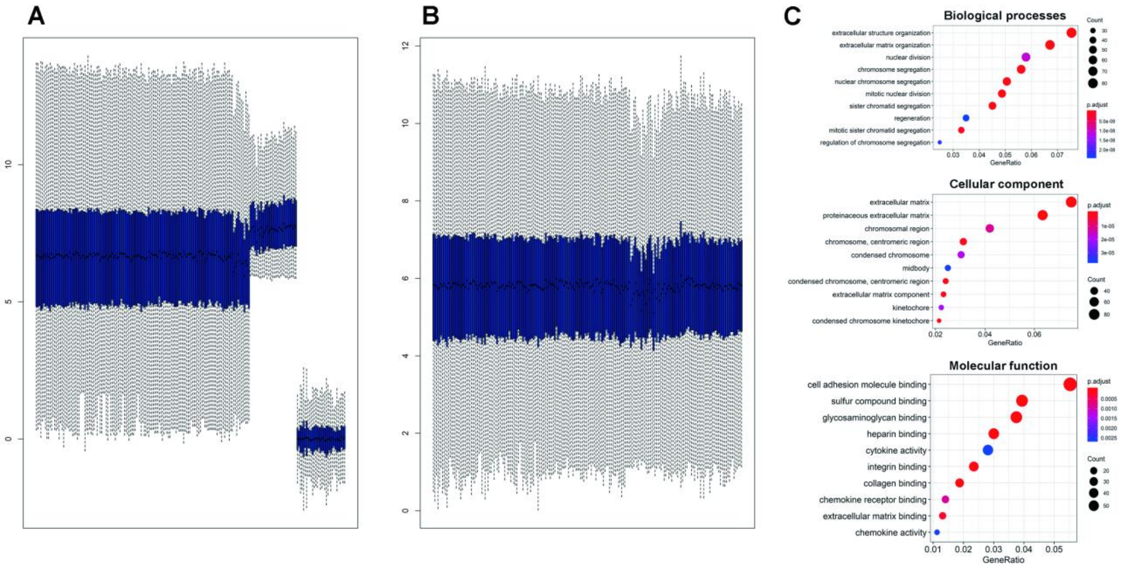


**Figure S1 (A)** Raw expression profiles of GSE45827, GSE50428, and GSE57297. **(B)** The expression profiles of GSE45827, GSE50428, and GSE57297 were normalized by R/SVA software package. **(C)** Gene Ontology (GO) analysis classified the DEGs into 3 groups, molecular function, biological process, and cellular component.

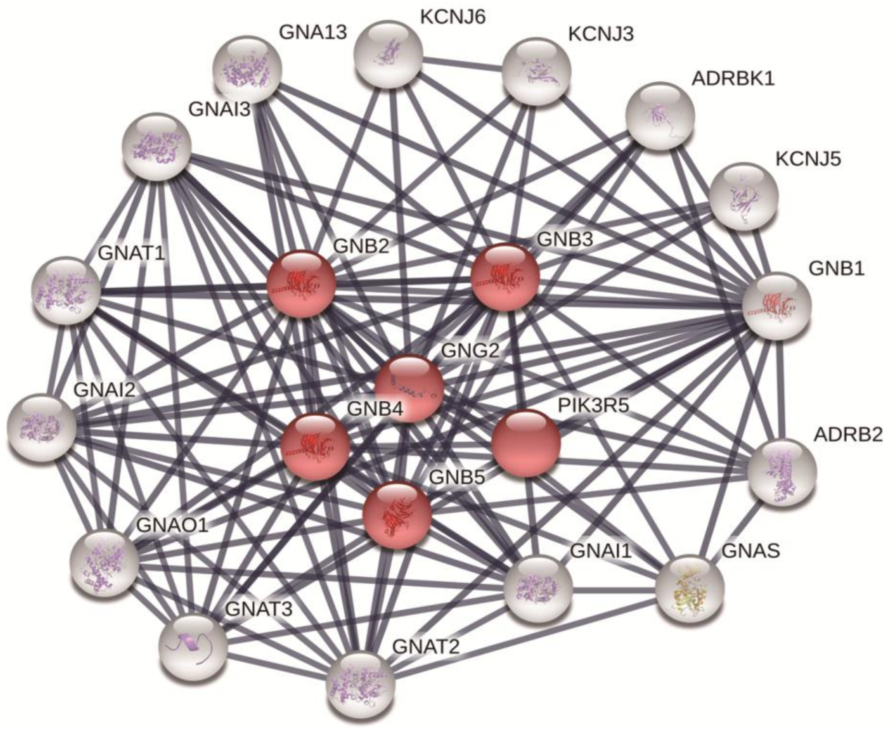


**Figure S2** Literature-based functional connectivity analysis of GNG2 was performed by STRING online tool. Red colors indicated the genes that were closely linked to PI3K-AKT signaling pathway.

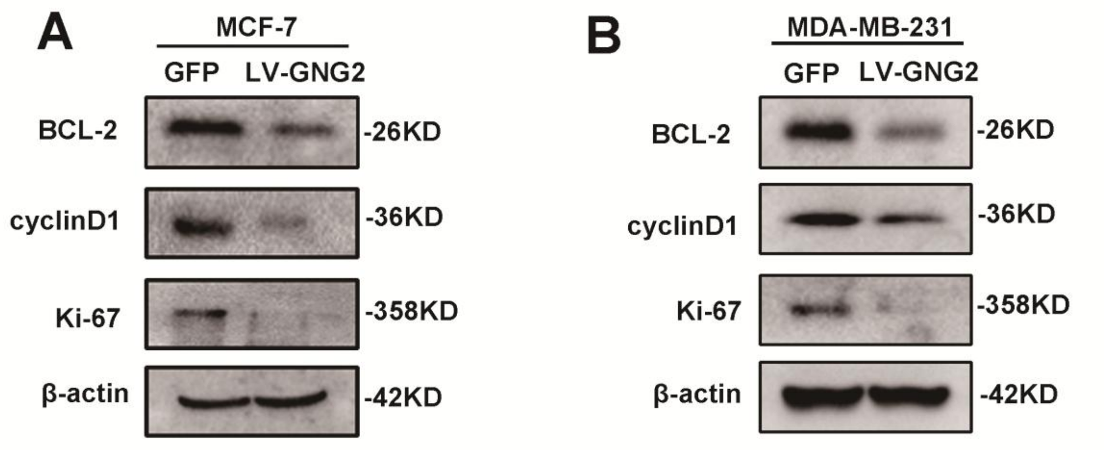


**Figure S3** Effects of GNG2 overexpression on proliferation- and apoptosis-related genes *in vitro*. MCF-7 **(A)** and MDA-MB-231 **(B)** cells were transfected with LV-*GFP* or LV-*GNG2* as described in the "Material and methods" section.

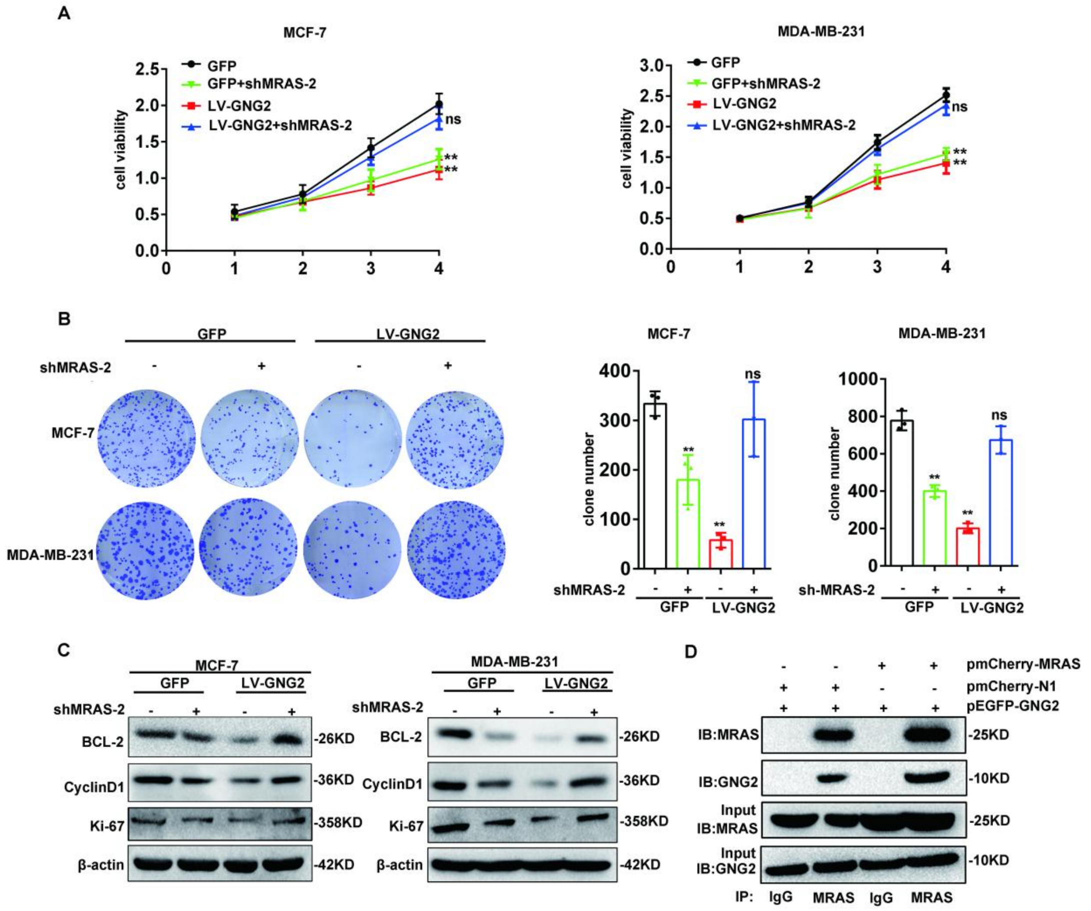


**Figure S4 Interaction between GNG2 and MRAS and the effect on tumor growth.** MCF-7 and MDA-MB-231 cells were transected with LV-*GNG2* or LV-*GFP*, or LV-*GNG2* + pGV-*shMRAS-2 or* LV-*GFP* + pGV-*shMRAS-2* as indicated in the methods. **(A)** Cell viability assay in MCF-7 (left) and MDA-MB-231 (right). **(B)** Cell proliferation experiments (left) and quantitative analysis (right). **(C)** Protein expression of proliferation- and apoptosis-related genes. **(D)** The interaction between GNG2 and MRAS in HEK-293T cells was examined by the Co-IP experiment followed by western blotting. Data are expressed as the mean ± SD. ** $p < 0.01$ *vs*. GFP.
