# Supplementary material for: GNG2 acts as a tumor suppressor in breast cancer through stimulating MRAS signaling
Source: Cell Death Dis. 2022 Mar 23;13(3):260. doi: 10.1038/s41419-022-04690-3 (PMC8943035; doi:10.1038/s41419-022-04690-3)

Original western blots:

Fig. 4A

GNG2 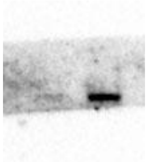

β-actin 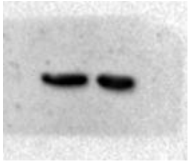

Fig.4B

GNG2 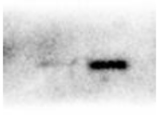

β-actin 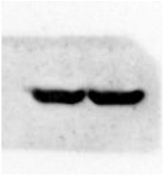

Fig.6B MCF-7

p-erk 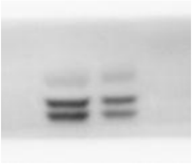

t-erk 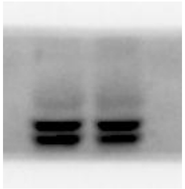

p-AKT

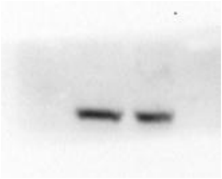

t-AKT

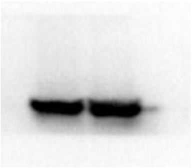

p-GSK3β

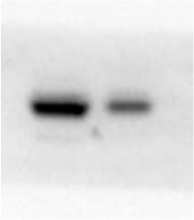

t-GSK3β

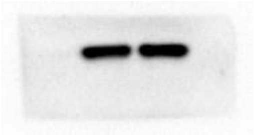

Fig.6B MDA-MB-231

p-erk

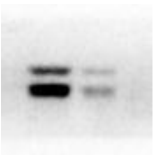

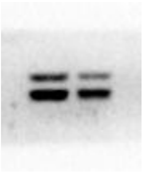

t-erk

p-AKT

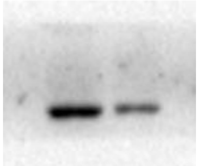

t-AKT

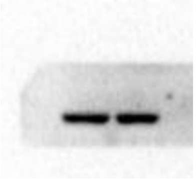

P-GSK3β

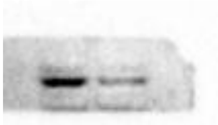

t-GSK3β

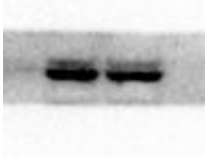

Fig.7D MCF-7

MRAS

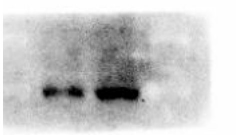

β-actin

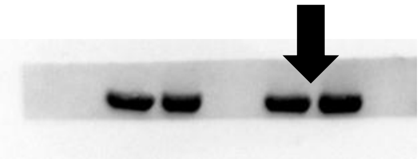

Fig.7D MDA-MB-231

MRAS

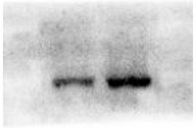

β-actin

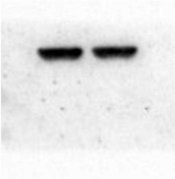

Fig.7E MCF-7

MRAS

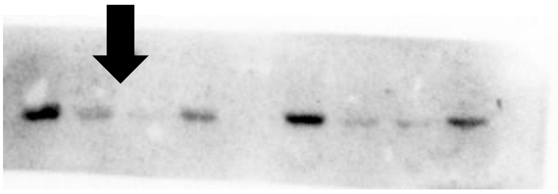

β-actin

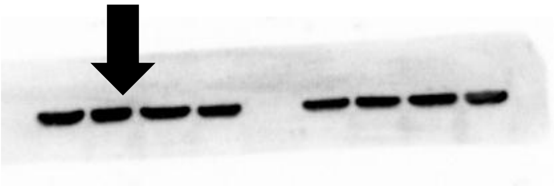

Fig.7E MDA-MB-231

MRAS

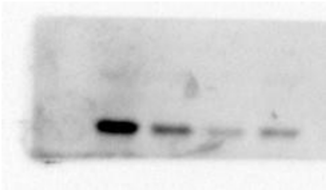

β-actin

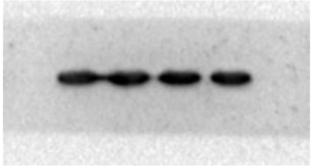

Fig.7F MCF-7

p-AKT

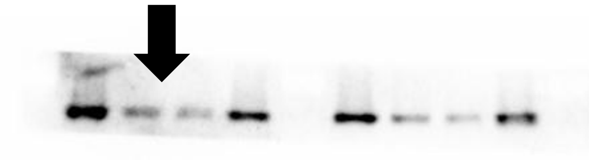

t-AKT

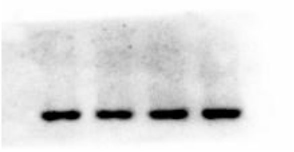

p-ERK

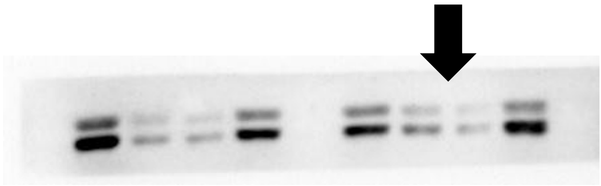

t-ERK

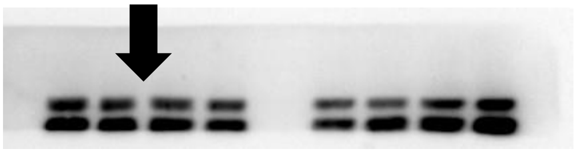

β-actin

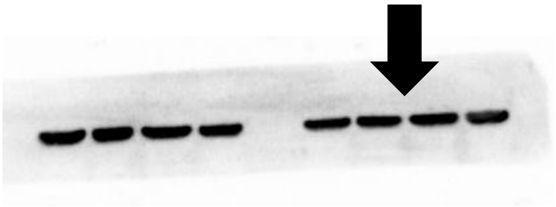

Fig.7F MDA-MB-231

p-AKT

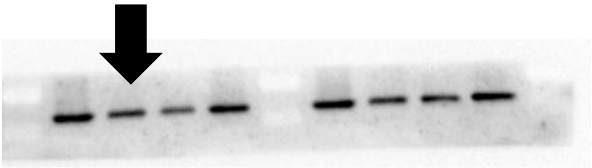

t-AKT

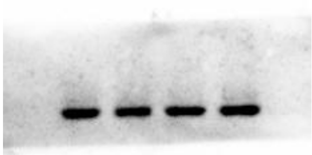

p-ERK

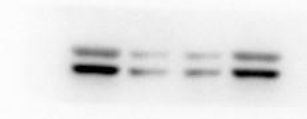

t-ERK

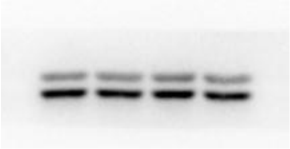

β-actin

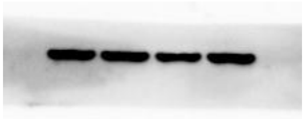

Fig.7H MCF-7

IB:MRAS

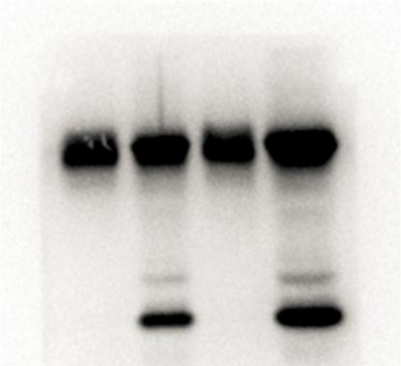

IB:GNG2

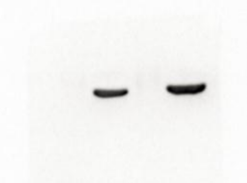

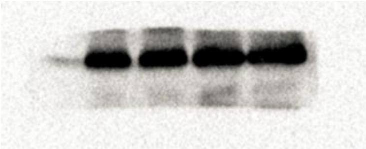

Input/IB:MRAS

Input/IB:GNG2

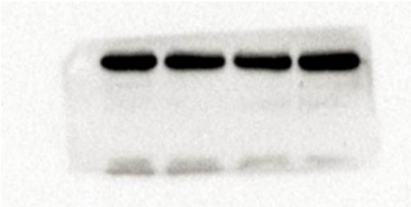

Supplementary material ：

figure S3A

BCL-2

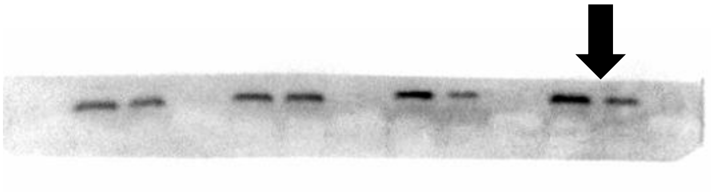

CyclinD1

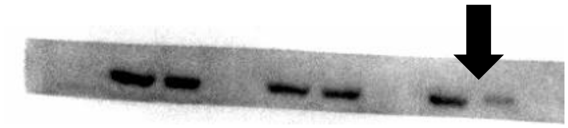

Ki-67

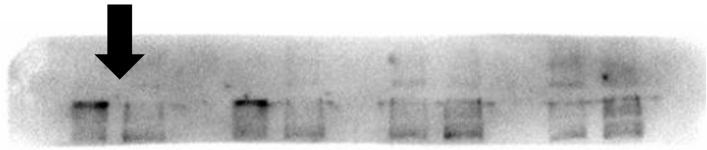

β-actin

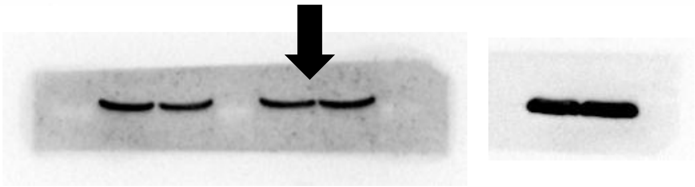

figure S3B

BCL-2

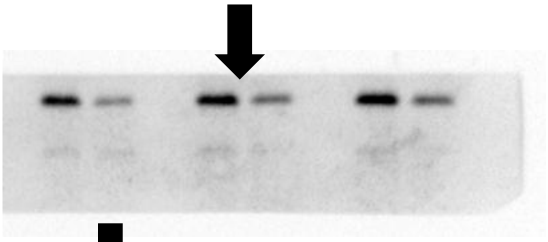

CyclinD1

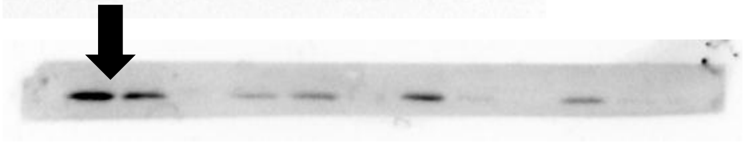

Ki-67

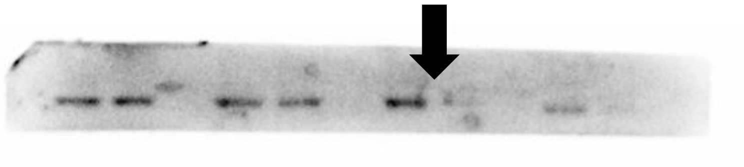

β-actin

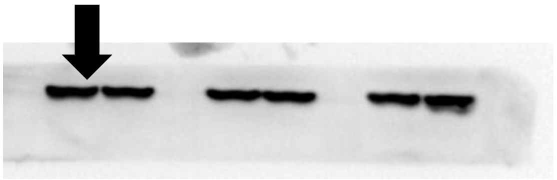

figure S4D　MCF-7

BCL-2

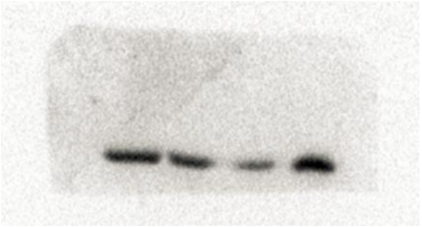

CyclinD1

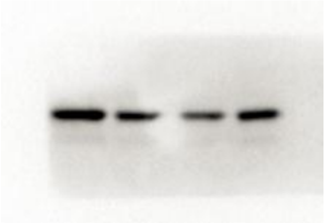

Ki67

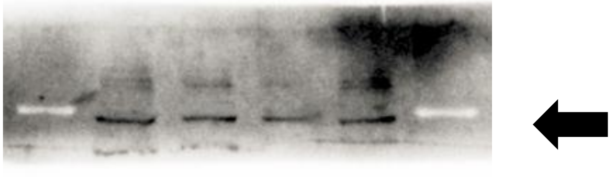

Actin

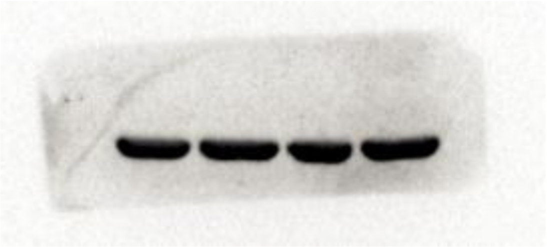

figure S4D MDA-MB-231

BCL2

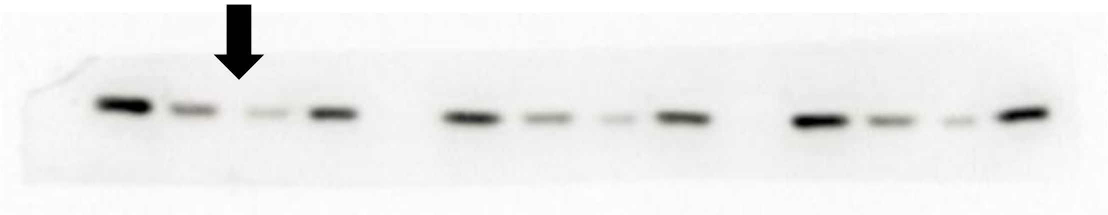

CyclinD1

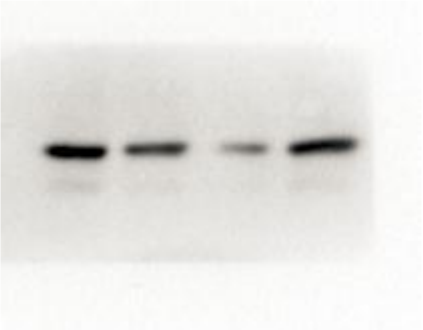

Ki67

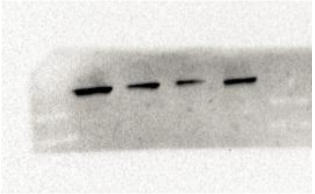

Actin

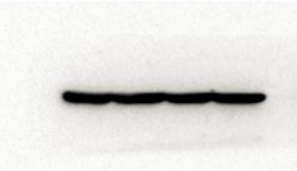

figure S4D 293T

IB:MRAS

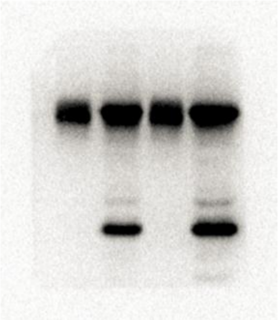

IB:GNG2

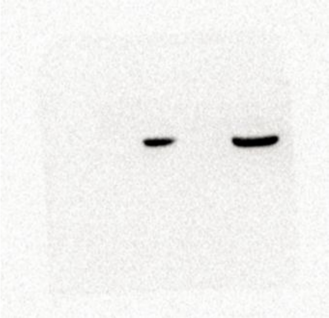

Input/IB:MRAS

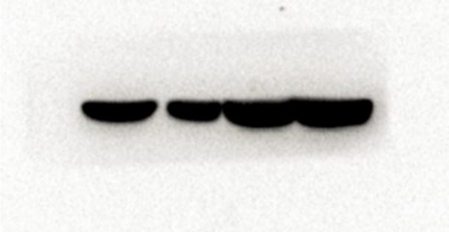

Input/IB:GNG2

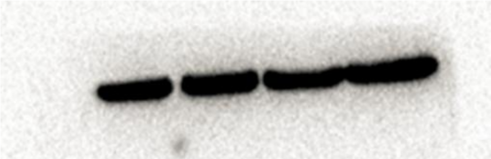

Supplement: Supplementary file 2 — Original Western Blots [file 41419_2022_4690_MOESM2_ESM.pdf]
